# Opposing roles of the entero-pancreatic hormone urocortin-3 in glucose metabolism in rats

**DOI:** 10.1007/s00125-022-05675-9

**Published:** 2022-03-24

**Authors:** Kaare V. Grunddal, Samuel A. J. Trammell, Cecilie Bæch-Laursen, Daniel B. Andersen, Stella F. S. Xu, Helle Andersen, Matthew P. Gillum, Seyed M. Ghiasi, Ivana Novak, Björn Tyrberg, Chien Li, Mette M. Rosenkilde, Bolette Hartmann, Jens J. Holst, Rune E. Kuhre

**Affiliations:** 1 Department of Biomedical Sciences, Faculty of Health and Medical, Sciences, University of Copenhagen, Copenhagen, Denmark; 2 Novo Nordisk Foundation Centre for Basic Metabolic Research, Faculty of Health and Medical Sciences, University of Copenhagen, Copenhagen, Denmark; 3 Global Obesity and Liver Disease Research, Novo Nordisk, Måløv, Denmark; 4 Department of Biology, Faculty of Science, University of Copenhagen, Copenhagen, Denmark; 5 Section of Cell Biology and Functional Genomics, Division of Diabetes, Endocrinology and Metabolism, Department of Metabolism, Digestion and Reproduction, Faculty of Medicine, Imperial College, London, UK; 6 Department of Physiology, Institute of Neuroscience and Physiology, Sahlgrenska Academy, University of Gothenburg, Gothenburg, Sweden; 7 Global Obesity and Liver Disease Research, Novo Nordisk, Seattle, WA USA

**Keywords:** Blood glucose, Corticotropin-releasing factor receptor 2, Gastric emptying, Glucose absorption, Insulin secretion, Somatostatin, Urocortin-3

## Abstract

**Aim/hypothesis:**

Urocortin-3 (UCN3) is a glucoregulatory peptide produced in the gut and pancreatic islets. The aim of this study was to clarify the acute effects of UCN3 on glucose regulation following an oral glucose challenge and to investigate the mechanisms involved.

**Methods:**

We studied the effect of UCN3 on blood glucose, gastric emptying, glucose absorption and secretion of gut and pancreatic hormones in male rats. To supplement these physiological studies, we mapped the expression of UCN3 and the UCN3-sensitive receptor, type 2 corticotropin-releasing factor receptor (CRHR2), by means of fluorescence in situ hybridisation and by gene expression analysis.

**Results:**

In rats, s.c. administration of UCN3 strongly inhibited gastric emptying and glucose absorption after oral administration of glucose. Direct inhibition of gastrointestinal motility may be responsible because UCN3’s cognate receptor, CRHR2, was detected in gastric submucosal plexus and in interstitial cells of Cajal. Despite inhibited glucose absorption, post-challenge blood glucose levels matched those of rats given vehicle in the low-dose UCN3 group, because UCN3 concomitantly inhibited insulin secretion. Higher UCN3 doses did not further inhibit gastric emptying, but the insulin inhibition progressed resulting in elevated post-challenge glucose and lipolysis. Incretin hormones and somatostatin (SST) secretion from isolated perfused rat small intestine was unaffected by UCN3 infusion; however, UCN3 infusion stimulated secretion of somatostatin from delta cells in the isolated perfused rat pancreas which, unlike alpha cells and beta cells, expressed *Crhr2*. Conversely, acute antagonism of CRHR2 signalling increased insulin secretion by reducing SST signalling. Consistent with these observations, acute drug-induced inhibition of CRHR2 signalling improved glucose tolerance in rats to a similar degree as administration of glucagon-like peptide-1. UCN3 also powerfully inhibited glucagon secretion from isolated perfused rat pancreas (perfused with 3.5 mmol/l glucose) in a SST-dependent manner, suggesting that UCN3 may be involved in glucose-induced inhibition of glucagon secretion.

**Conclusions/interpretation:**

Our combined data indicate that UCN3 is an important glucoregulatory hormone that acts through regulation of gastrointestinal and pancreatic functions.

**Graphical abstract:**

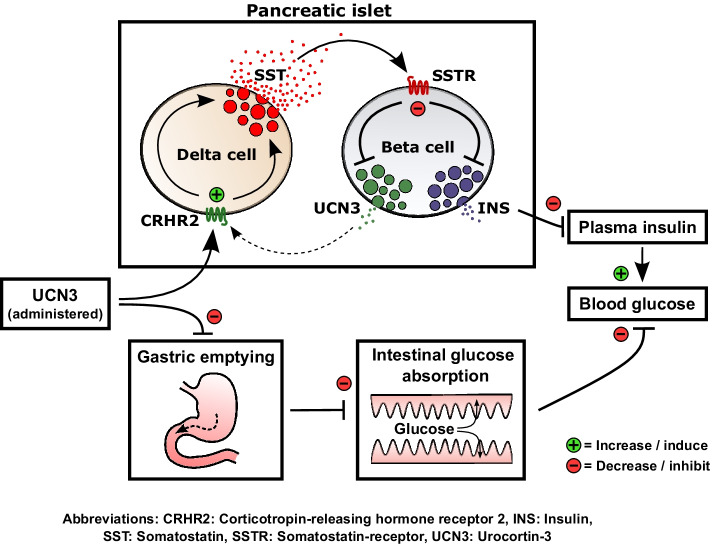

**Supplementary Information:**

The online version contains supplementary material available at 10.1007/s00125-022-05675-9.

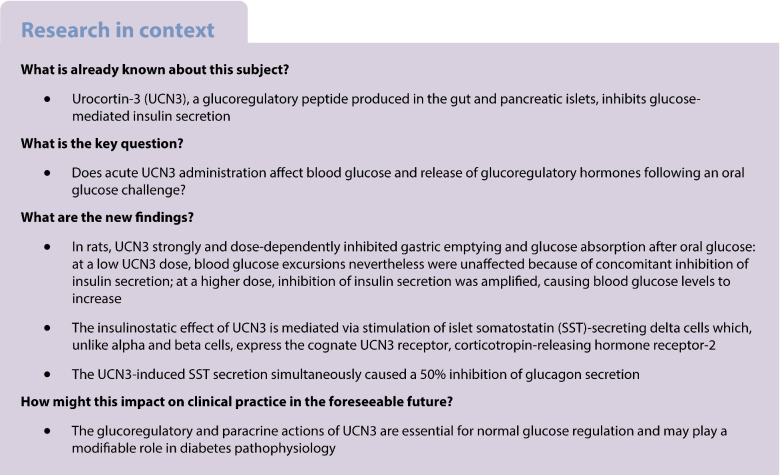



## Introduction

Urocortin-3 (UCN3), a 38-amino-acid peptide from the corticotropin-releasing factor family [1, 2], is expressed in beta cells and is co-secreted with insulin [3]. In mice and rats, UCN3 administration impairs glucose tolerance during OGTT [3–5] by mechanisms that may involve altering secretion of glucoregulatory hormones from the pancreatic islets, although observations conflict. Some studies in rats showed that UCN3 stimulated glucagon and insulin release in vivo and from isolated islets [3, 5], whereas another showed that UCN3 inhibits insulin secretion by stimulation of islet somatostatin (SST) release [4]. In addition, UCN3 has been shown to inhibit, via unclear mechanisms, gastric emptying [6, 7], which would reduce postprandial blood glucose excursions. Collectively, these data suggest that UCN3 may be an important yet underappreciated regulator of postprandial metabolism [8]. However, many fundamental aspects of UCN3 physiology remain unclear, including the dose-dependency of its opposing effects on post-challenge glucose tolerance (inhibition of gastric emptying and inhibition of insulin secretion). Here, we further clarify the effects and the dose-dependency of UCN3 on glucose excursions, gastric emptying, glucose absorption and lipolysis in rats challenged orally with glucose. Furthermore, we investigated the mechanisms underlying UCN3’s glucoregulatory effects.

## Methods

### Animals and ethical considerations

Male rats were purchased from Janvier (Saint Berthevin Cedex, France). Experiments were approved by the Danish Animal Experiments Inspectorate (2018-15-0201-01397 and 2020-15-0201-00756). For further details, see electronic supplementary material (ESM) Methods.

### In vivo study 1

Rats fasted for 8–12 h were used. Blood was sampled from conscious rats by sublingual puncture (200 μl/sample) into EDTA-coated tubes, which were immediately placed on ice. After collection of baseline samples (at −10 min and −5 min, plotted as time 0 min), rats received glucose by oral gavage (2 g/kg; containing in addition paracetamol [acetaminophen] 100 mg/kg [25 mg/ml]) at time 0 min together with a subcutaneous injection (200 μl) of one of the following compounds prepared in isotonic saline (154 mmol/l NaCl) with 0.1% HSA: isotonic saline (vehicle); UCN3 (30 nmol/kg); glucagon-like peptide-1 (GLP-1; 30 nmol/kg; positive control); or the corticotropin-releasing hormone receptor 2 (CRHR2) antagonists, antisauvagine-30 or K41498 (100 μg/kg). Plasma exposure data later showed that the UNC3 group had ~¼ of the concentration observed in the 30 nmol/kg group in the second in vivo study (where concentrations after formulation was verified). Concentration is thus presented as an adjusted dose of 7.5 nmol/kg. Blood was collected at time points 5, 10, 15, 30 and 45 min by the procedure described above. Samples were centrifuged (1650*g*, 4°C, 10 min) within 30 min and plasma was stored at −20°C. At the end of the experiments, rats were returned to their housing location and were observed daily for a week. No signs of failure to thrive were observed. Weight did not differ between experimental groups (~350 g, *p*>0.58 for all groups).

### In vivo study 2

The study design was as for study 1 but with following exceptions: 250 μl blood was withdrawn at each time point; time points were −30, −15 (baseline), 5, 10, 15, 30, 45 and 90 min; UCN3 challenge at time 0 min was with 2, 7.5 or 30 nmol/kg; and rats were euthanised at the end of the study (by CO_2_ overdose). UCN3 was formulated in 50 mmol/l HEPES, 180 mmol/l glycerol and 0.007% polysorbate 20, pH 8. Weight did not differ between experimental groups (~350 g, *p≥*0.74 for all groups).

### UCN3 t½ in rats

Plasma samples from in vivo study 1 and 2 were used to determine UCN3 t½. For further details, see ESM Methods.

### Perfusion studies

Rat small intestines and pancreases were isolated in situ by cannulation of the relevant vasculature. Organs were perfused with a physiological buffer and samples were collected each minute. For detailed description of procedures, see ESM Methods.

### Pharmacology studies

COS-7 cells were modified for either mouse or rat CRHR2 expression using a transient calcium phosphate precipitation transfection procedure [9] with pCMV6-Entry vectors containing untagged *Crhr2* clones for either the rat or mouse version of the receptor (catalogue no. RN206899 and MC208334; OriGene Europe, Herford, Germany). Two days after transfection, cells were incubated either with synthetic mouse UCN3 at a concentration range of 1×10^−12^ to 1×10^−7^ mol/l or with one of the following CRHR2 antagonists: antisauvagine-30; or K41498. Changes in receptor activity were quantified by use of in vitro HitHunter cAMP assay. For further details, see ESM Methods.

### UCN3 concentrations in rat gut and pancreas

Protein concentrations were quantified in gastrointestinal and pancreas tissue biopsies (~1–1.5 cm/piece) by Pierce BCA Protein Assay Kit (catalogue no. 23227; Thermo Fisher Scientific, USA) and extracts were purified using tc18 cartridges (catalogue no. 036810; Waters, USA). See ESM Methods for further information.

### In situ hybridisation

Rat (male Wistar) pancreas, gastric ventricle, jejunum, distal ileum and mid-colon were investigated for *Ucn3* and *Crhr2* expression by standard procedures, using antibodies listed in ESM Table 1 and probes listed in ESM Table 2.

### Expression analysis of UCN3 and CRHR2 in rat and human islets

Expression of genes encoding UCN3 and CRHR2 was investigated in rat islets by qPCR and in human islets by re-analysing publicly available human islet single-cell sequencing data. The qPCR used primers listed in ESM Table 3. The expression of *Ucn3* and *Crhr2* was compared with expression of the well-known genes *Gcg*, *Glp-1r* (also known as *Glp1r*), *Ins-1* (also known as *Ins1*) and *Ins-2* (also known as *Ins2*) in rat islets. The expression of *UCN3* and *CRHR2* was compared with expression of *GCG*, *UCN1*, *UCN2* and *GLP1R* in human islet alpha, beta and delta cells. Further information can be found in ESM Methods.

### Biochemical measurements

Quantifications were done by either in-house RIA or by use of commercially available assays. Samples were assayed in a non-randomised manner. For further information about the assays and procedures, see ESM Methods and ESM Table 4. To quantify plasma 3-*O*-methyl-*d*-glucose (3-OMG) concentrations, we developed a sensitive and low volume LC/MS-based quantification (see ESM Methods for further information). Samples were also in this case assayed in a non-randomised manner.

### Data presentation and statistical analysis

Data are expressed as means ± SEM. For perfusion data, averaged basal and response outputs were calculated by averaging output values over the entire stimulation period (response, 15 min) and the period leading up to stimulus administration (15 min, baseline). Output (fmol/min) was the product of peptide concentration (pmol/l) and flow rate (ml/min). Statistical significance was assessed by two-way ANOVA or by one-way ANOVA for repeated measurements (as indicated in figure legends), followed by Tukey’s multiple comparison test. Alternatively, Student’s paired *t *test was used when applicable. Statistical tests were performed in GraphPad Prism (vs. 9; La Jolla, CA, USA). Graphs were constructed in GraphPad Prism and edited in Adobe Illustrator (vs. 2021; San Jose, CA, USA). *p*<0.05 was considered significant. No animals/samples were excluded from the presented data set.

## Results

### Effects of UCN3 on blood glucose, gastric emptying and glucose absorption

**Fig. 1:**
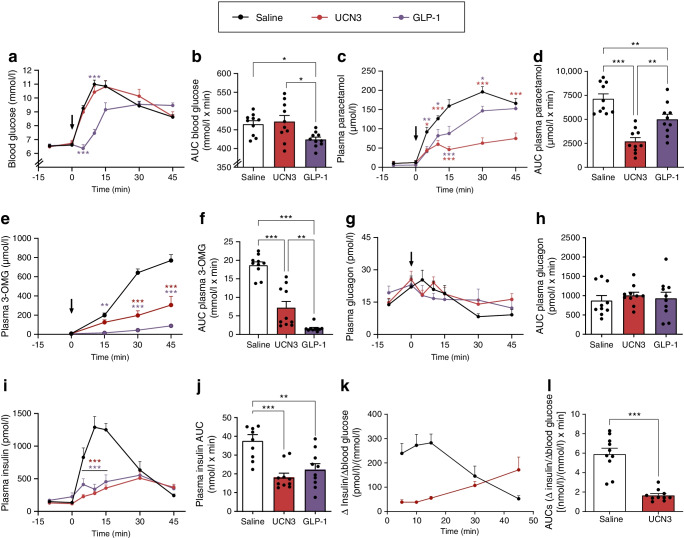
Effects of UCN3 on blood glucose, gastric emptying and glucose-stimulated secretion of insulin and glucagon in the rat. Response to an oral challenge with glucose (2 g/kg) in combination with s.c. injection of vehicle, UCN3 (adjusted dose 7.5 nmol/kg) or GLP-1 (30 nmol/kg, positive control). Glucose and s.c. stimuli were both administered at 0 min (indicated by arrow). Baseline samples were taken at −10 min and 0 min. Plots of variables over time are shown, with respective AUCs, for blood glucose (**a**, **b**), plasma paracetamol (**c**, **b**), plasma 3-OMG (**e**, **f**), plasma glucagon (**g**, **h**), plasma insulin (**i**, **j**) and ∆insulin/∆blood glucose ratio (**k**, **l**). Data are shown as means + SEM, *n* = 10. *p<0.05, **p<0.01 and ***p<0.001 (two-way ANOVA followed by Tukey’s multiple comparison test, testing groups against vehicle [**a**, **c**, **e**, **g**, **i**], one-way ANOVA followed by Tukey’s multiple comparison test [**b**, **d**, **f**, **h**, **j**] or, in l, paired *t* test). ∆insulin values in (**k**) and (**l**) were calculated by subtracting baseline values (mean of −10 min and 0 min values) from subsequent values. AUCs covered −5 min to 45 min (**b**, **d**, **h**, **j**), 5–45 min (**f**) or 5–30 min (**l**). Arrows indicate time point of OGTT and compound administration

Baseline blood glucose was ~6.5 mmol/l (*p*≥0.80 between groups). Blood glucose profiles in the vehicle- and UCN3-treated groups were similar (*n*=10) (individual time points: *p*=0.47–0.99 and AUC *p*>0.99), while GLP-1 injection resulted in an expected pronounced delay in blood glucose rise (Fig. 1a,b). A similar delay in glucose increase was seen after administration of antisauvagine-30 + K41498, with significantly lower glucose AUCs compared with vehicle (*p*<0.05, ESM Fig. 1). Compared with the vehicle-treated group, post-challenge paracetamol concentrations were 50–80% lower at 5, 10, 15 and 30 min in both the GLP-1 and the UCN3 group (*p*<0.05 to *p*<0.0001, Fig. 1c). Paracetamol AUC was 60% lower and 25% lower in UCN3 and GLP-1 groups compared with the vehicle group (Fig. 1d). The AUC for plasma 3-*O*-methyl-d-glucose (3-OMG) (a marker of glucose absorption [10]) amounted to only 30–50% and 10–20% of the vehicle group, respectively (Fig. 1f), consistent with marked inhibition of gastric emptying.

### Effects of UCN3 on blood glucose regulating hormones

Plasma glucagon concentrations at baseline were similar between all groups (Fig. 1g) and administration of 30 nmol/kg UCN3 did not affect AUCs compared with administration of vehicle (Fig. 1h).

Plasma insulin concentrations at baseline did not differ between groups (*p*≥0.99, *n*=10, Fig. 1i). The insulin response to the OGTT was delayed and considerably blunted in the UCN3 group (adjusted dose: 7.5 nmol/kg) compared with the vehicle group, amounting to less than half of that in the vehicle group. (Fig. 1i,j). Thus, the ∆insulin/∆glucose ratio was overall ~70% lower in the UCN3 group compared with the vehicle group (Fig. 1k,l).

### Dose-dependent effects of UCN3 on blood glucose, gastric emptying, glucose absorption, lipolysis and plasma concentrations of insulin

**Fig. 2:**
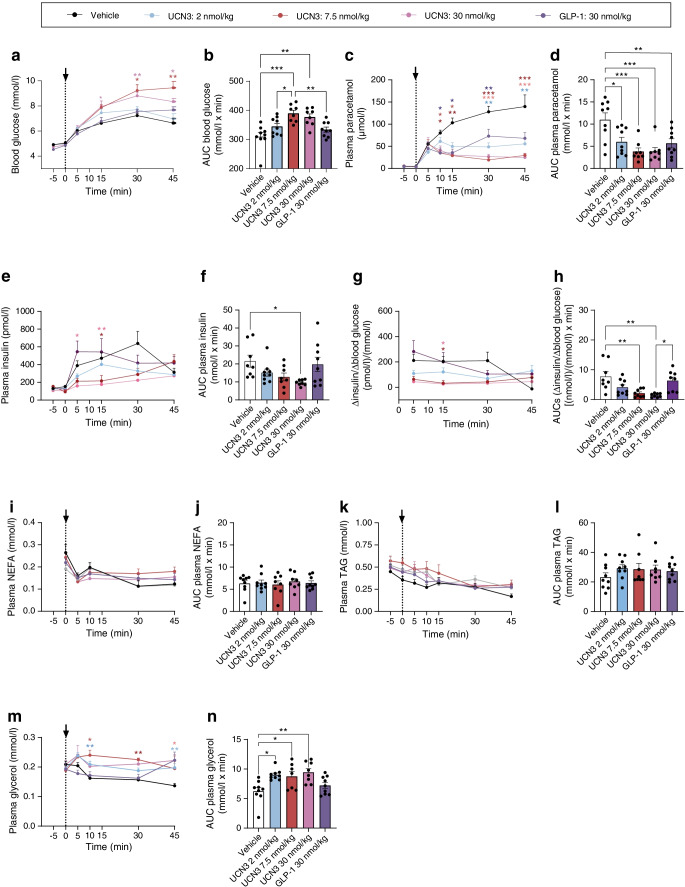
Dose-dependent effects of UCN3 on blood glucose, gastric emptying, glucose-stimulated secretion of insulin and lipolysis in the rat. Response to an oral glucose (2 g/kg) challenge in combination with s.c. injection of either vehicle (control), three different doses of UCN3 (2, 7.5 and 30 nmol/kg), or GLP-1 (7–36amide, 30 nmol/kg). Plots of variables over time are shown, with respective AUCs, for blood glucose (**a**, **b**), plasma paracetamol (**c**, **d**), plasma insulin (**e**, **f**), ∆insulin/∆blood glucose (**g**, **h**), plasma NEFA (**i**, **j**), plasma TAG (**k**, **l**) and plasma glycerol (**m**, **n**). AUCs covered −5 min to 45 min (**b**, **d**, **f**, **j**, **l** and **n**) or 5–45 min (**h**). Baseline sample points taken at −30 and −15 min were in all cases plotted as −5 min and 0 min. Data are shown as means + SEM, *n* = 8 or 9. *p<0.05, **p<0.01 and ***p<0.001 (two-way ANOVA followed by Tukey’s multiple comparison test, testing groups against vehicle [**a**, **c**, **e**, **g**, **i**, **k**, **m**] or by one-way ANOVA followed by Tukey’s multiple comparison test [**b**, **d**, **f**, **h**, **j**, **l**, **n**]). TAG, triacylglycerol. Arrows indicate time point of OGTT and compound administration

To investigate the dose-dependency of UCN3’s effects on post-challenge blood glucose levels as well as its inhibitory effects on gastric emptying and insulin secretion, we next used three different doses of UCN3 (2, 7.5 and 30 nmol/kg). With the low UCN3 dose (2 nmol/kg), blood glucose and paracetamol data matched those in the first in vivo study (Fig. 2a–d). This was presumably due to concomitant inhibition of insulin secretion, which at this lower dose was variable in effect size and not significant (Fig. 2e,f). At the medium and high UCN3 doses (7.5 and 30 nmol/kg), blood glucose levels were higher than in either the vehicle group or low-dose UCN3 group, with the AUC (−5 min to 45 min) being increased by ~20% (*p*<0.05, Fig. 2a,b); no statistically significant differences were observed between the 7.5 and 30 nmol/kg groups. The dose-dependent differences in net effect on post-challenge blood glucose likely resulted from a combination of comparable inhibitory effect on gastric emptying (as measured by plasma paracetamol concentrations) at the low, medium and high dose (AUC for 2 vs 7.5 nmol/kg UCN3, *p*=0.62; AUC for 2 vs 30 nmol/kg UCN3, *p*=0.63; Fig. 2c,d) and stronger and significant inhibition of insulin secretion at the high dose (AUCs [−5 min to 45 min] for vehicle 21.6±3.34 nmol/l × min vs 100 nmol/kg UCN3 9.60±0.57 nmol/l × min; *p*<0.05; Fig. 2e,f). As a reflection of this, the ∆insulin/∆glucose ratio dose-dependently decreased. AUCs (−5 min to 45 min) in the 2, 7.5 and 30 nmol/kg UNC3 groups thus only amounted to, respectively, ~40%, ~28% and ~20% of that of vehicle (Fig. 2g,h). We also investigated whether UCN3 affected lipolysis. Neither NEFA nor triacylglycerol was affected at any of the UCN3 doses (Fig. 2i–l) but plasma glycerol increased significantly with all UCN3 doses (*p*<0.05 vs vehicle) (Fig. 2m,n). A time point at 90 min was also included in the experiment but this was subsequently judged irrelevant due to minimal UCN3 exposure (ESM Fig. 3). However, even including the point at 90 min, the response patterns were largely the same. An exception to this was plasma insulin which, in this case, was not significantly different in the UCN3 groups compared with vehicle (ESM Fig. 2).

### UCN3 t½ in rats

**Fig. 3:**
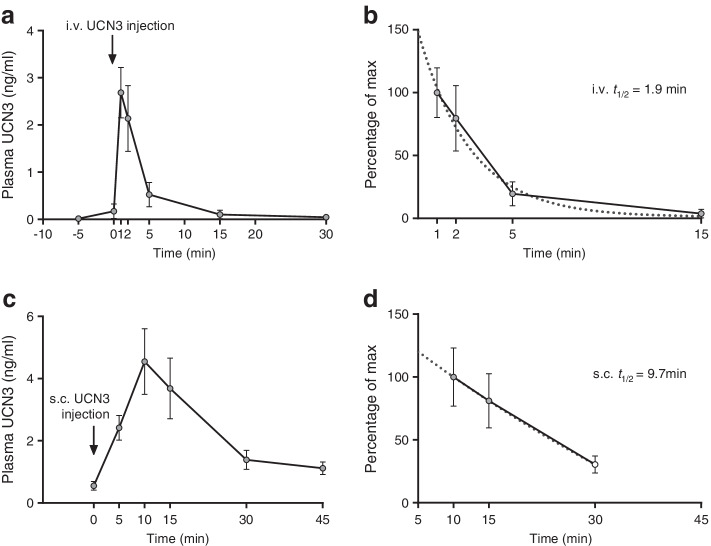
*t*_½_ of UCN3 in the rat. Response to i.v. (via vena cava inferior, 2 nmol/kg) or s.c. injection of UCN3 (7.5 nmol/kg). (**a**, **c**) Plasma UCN3 concentrations over time following i.v. (**a**) or s.c. (**c**) injection. (**b**) Data from **a** (at the 1 min point) and (**d**) data from **c** (at the 10 min point) expressed as percentage of maximum concentration. *t*_½_ was calculated by one-phase linear regression as indicated in (**b**) and (**d**). Regression fits are indicated by dotted lines. Data are shown as means ± SEM, *n* = 4 (**a**, **b**) or 10 (**c**, **d**). Arrows indicate time point of compound administration

The t½ of UCN3 in rodents is unknown, so we determined the t½ of UCN3 after i.v. and s.c. administration in rats. The t½ after i.v. and s.c. administration was 1.9 and 12.2 min (based on first-order kinetics), respectively (Fig. 3a–d). To compare plasma exposure between experiments, we also determined plasma concentrations in rats receiving a dose of 30 nmol/kg UCN3 in the second in vivo study (Fig. 2). Data are shown in ESM Fig. 3.

### Direct effects of UCN3 secretion on glucose-dependent insulinotropic polypeptide, GLP-1, neurotensin and SST from isolated perfused rat small intestine

**Fig. 4:**
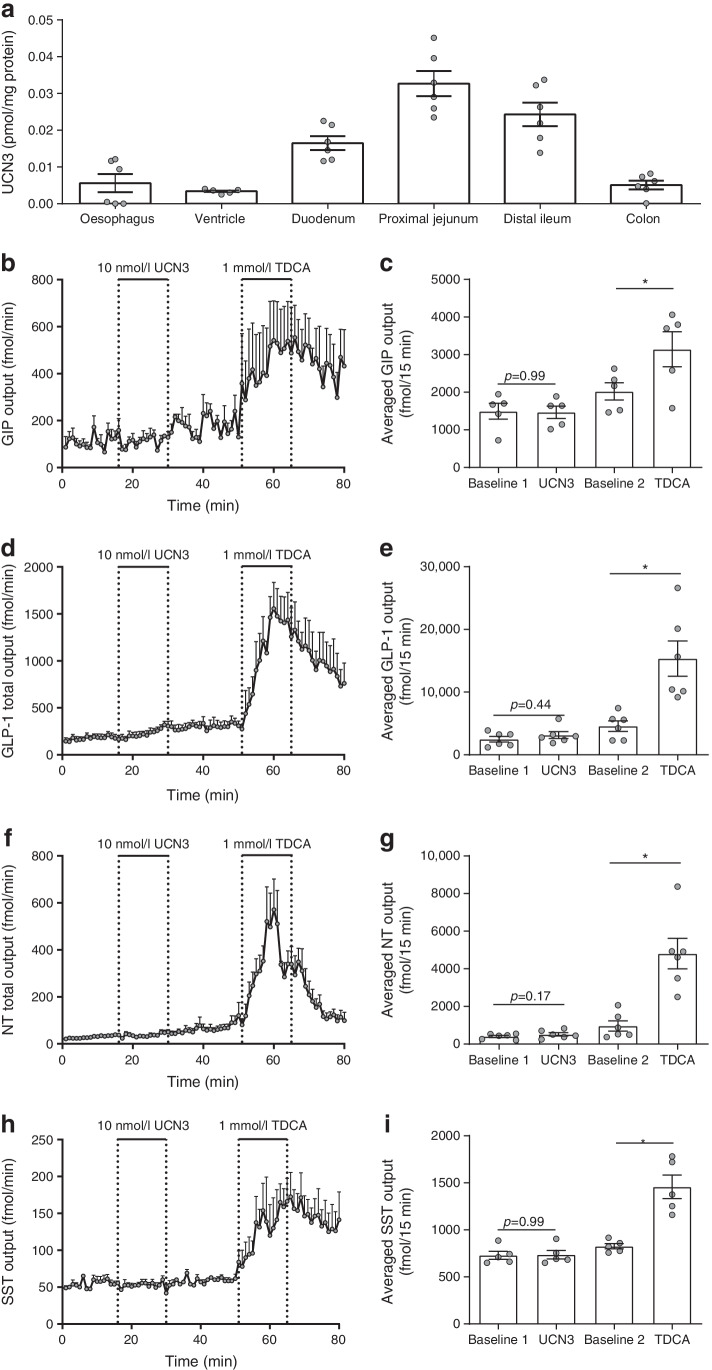
Concentrations of UCN3 along the rat intestine and effects of UCN3 on secretion of glucoregulatory hormones from isolated perfused rat small intestines. (**a**) Extractable UCN3 concentrations in rat oesophagus to colon. (**b**–**i**) Secretion of GIP (**b**, **c**), GLP-1 (**d**, **e**), NT (**f**, **g**) and SST (**h**, **i**) in response to arterial infusions of UCN3 (10 nmol/l) or taurodeoxycholic acid (1 mmol/l, positive control). Secretion is plotted as min–min outputs and as respective average total output during the stimulation periods (15 min). Data are presented as means ± SEM, *n* = 6. *p<0.05 (one-way ANOVA for repeated measurements followed by Tukey post hoc test). TDCA, taurodeoxycholic acid

To test whether UCN3 acts locally in the gut to indirectly inhibit gastric emptying via secretion of one or more gut hormones, we first examined UCN3 concentrations along the rat gastrointestinal tract. UCN3 concentrations above the detection limit (0.14 ng/ml) were found in extracts of all investigated segments; concentrations were highest in the proximal jejunum and distal ileum (Fig. 4a, *n*=6). A similar distribution pattern was observed when data were normalised to tissue wet weight (data not shown). Subsequently, we investigated whether intra-arterial UCN3 infusion affected glucose-dependent insulinotropic polypeptide (GIP), GLP-1, neurotensin (NT) or SST secretion from isolated perfused rat small intestine. We found that this was not the case, although taurodeoxycholic acid (positive control) increased their secretion at least twofold (*p*<0.05, *n*=6, Fig. 4b–i). This suggests that UCN3 could interact directly with the stomach and intestine, which would require CRHR2 expression in these organs.

### *Crhr2* expression in the rat gastrointestinal tract

**Fig. 5:**
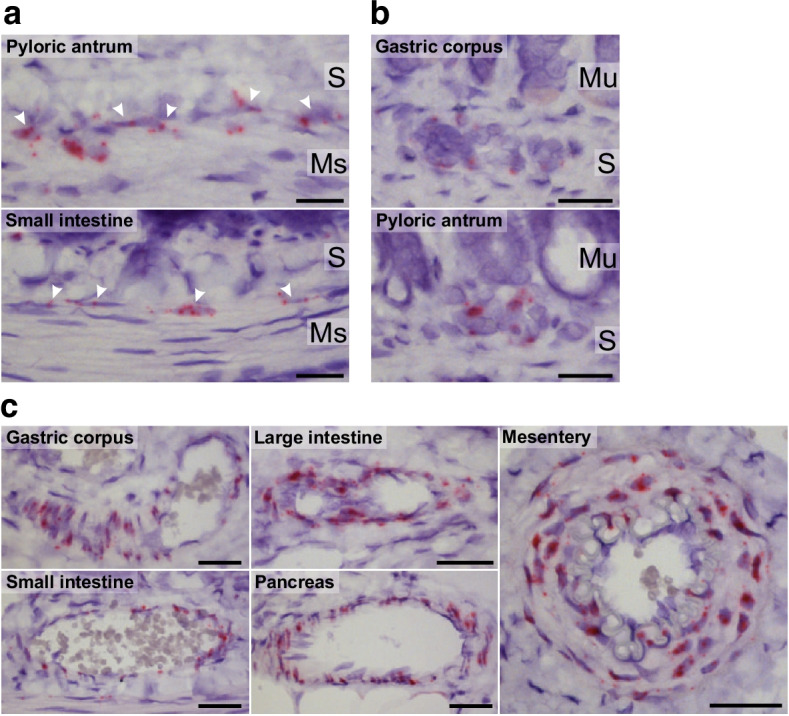
*Crhr2* expression in the rat gastrointestinal tract. Representative images of chromogenic in situ hybridisation staining of *Crhr2* mRNA (red) in interstitial cells of Cajal along the submucosal surface of the circular smooth muscle layer in the pyloric antrum and the small intestine (**a**), submucous plexus of the gastric corpus and pyloric antrum (**b**) and smooth muscle layer of arterioles in the gastric corpus, small and large intestine, pancreas, and mesentery (**c**). Nuclei were counterstained with haematoxylin. Scale bar, 20 μm (**a**, **b**) or 30 μm (**c**). *n* = 3 (male rats). Ms, muscularis propria; Mu, mucosa; S, submucosa

*Crhr2* transcripts were found in cells along the submucosal surface of the circular muscle layer in the pyloric antrum, jejunum and distal ileum, morphologically resembling intestinal cells of Cajal (Fig. 5a), and in the stomach in the submucous nerve plexus of the gastric corpus and in the pyloric part of the antrum (Fig. 5b) (data from positive and negative controls studies are shown in ESM Fig. 4). *Crhr2* expression was rich in the smooth muscle layer of arterioles in the stomach, small and large intestine, pancreas and mesentery (Fig. 5c) but the gastrointestinal mucosa ranging from the stomach to the colon was devoid of staining, except for very few and scattered transcripts within the lamina propria (data not shown).

### *Ucn3* and *Crhr2* expression in rat and human islets

**Fig. 6:**
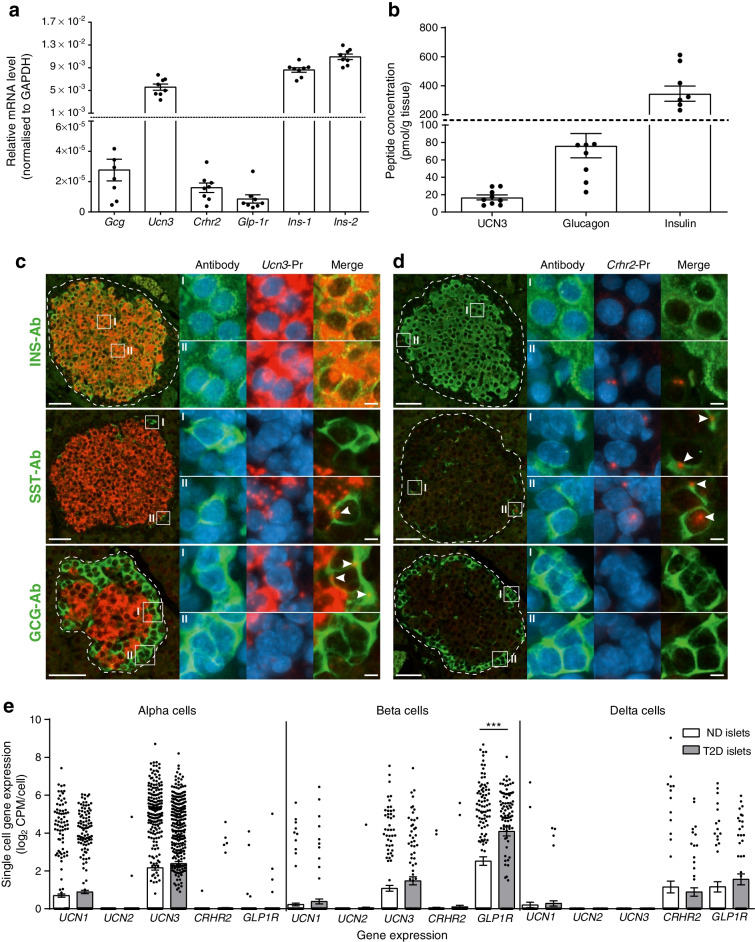
Extractable UCN3, glucagon and insulin concentrations in rat pancreas and expression of *Gcg*, *Ucn3* and *Crhr2* in rat islets and in islets from human donors with or without type 2 diabetes. (**a**) Expression of *Gcg*, *Ucn3*, *Crhr2*, *Glp-1r*, *Ins-1* and *Ins-2* in rat islets (quantified by real-time quantitative PCR). *n* = 8. (**b**) Concentrations of extractable UCN3, glucagon and insulin in rat pancreas. *n* = 9. (**c**, **d**) Representative images of fluorescence in situ hybridisations of pancreatic islets stained for *Ucn3* (**c**) or *Crhr2* (**d**) mRNA (red) and cells immunofluorescence-stained with antibodies against insulin, glucagon and SST (green) with blown-up areas indicated by I and II. Arrowheads indicate overlap between peptide hormone staining and mRNA staining. Nuclei are counterstained with DAPI (blue). Scale bar, 50 μm (5 μm in blown-up image). *n* = 3 male rats. (**e**) Single-cell expression of genes encoding *UCN1–3*, *CRHR2* and *GLP1R* in human islets from donors with or without type 2 diabetes. Circles show expression in respective single cells (no. of cells from non-diabetic/diabetic donors: alpha cells 443/443; beta cells 171/99 and delta cells 59/55) and bars indicate mean ± SEM expression levels in respective cell populations. Cells with a read count per million kilobases (RPKM) > 0 were considered positive. Data are shown as means ± SEM, *n* = 6 islets (non-diabetic) or 4 islets (diabetic). ***p<0.001 (one-way ANOVA followed by Tukey post hoc test). Ab, antibodies; GCG, glucagon; INS, insulin; ND, non-diabetic; Pr, probes; T2D, type 2 diabetes

*Ucn3* was highly expressed in the rat pancreas and amounted to 20–50% of the expression of *Ins-1* and *Ins-2* (Fig. 6a, *n*=8). The expression translated into high concentrations of extractable UCN3, well above the detection limit (pmol/g tissue: glucagon 76.3±14.0; UCN3 16.9±2.86; insulin 345±52.1, *n*=9, Fig. 6b). In situ hybridisation studies showed that *Ucn3* transcripts were highly abundant in insulin-containing beta cells but not in glucagon-containing alpha cells or SST-producing delta cells (Fig. 6c). *Crhr2* was expressed in rat islets at a level similar to that of *Glp-1r* (Fig. 6a) and, based on in situ hybridisation, the expression was exclusively observed in delta cells (Fig. 6d) (data from positive and negative controls studies are shown in ESM Fig. 5).

To investigate whether these expression patterns translated to human islets, we investigated single-cell expression of *UCN3* and *CRHR2* as well as *UCN1*, *UCN2* and *GLP1R* in human alpha cells, beta cells and delta cells from non-diabetic donors and donors with type 2 diabetes by re-analysing publicly available raw RNA sequencing data [11] (Fig. 6e). *CRHR2* was exclusively expressed by delta cells at a level comparable to delta-cell expression of *GLP1R*. *UCN3* was detected in both beta cells and alpha cells. *UCN1* and *UCN2* expression was minimal or non-detectable (Fig. 6e). *GLP1R* was upregulated in beta cells from individuals with type-2-diabetes (*p*<0.0001) whereas the remaining genes were not differentially expressed (*p*>0.05).

### Effects of UCN3 and CRHR2 on glucagon, insulin and SST secretion from isolated perfused rat pancreases

**Fig. 7:**
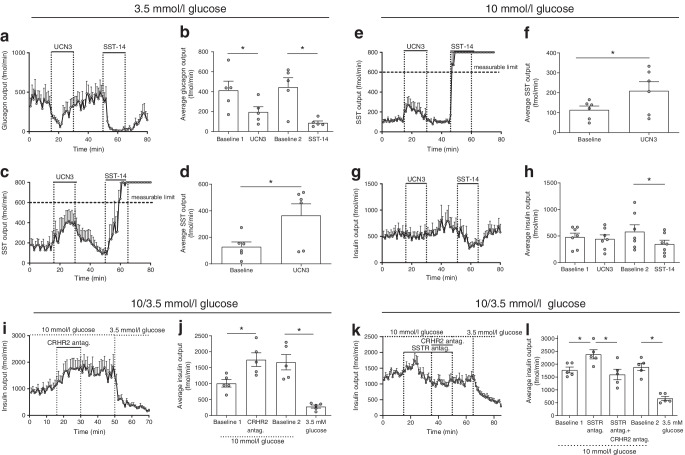
Effects of UCN3 infusion and blockage of CRHR2 signalling on secretion of glucagon, insulin and SST from isolated perfused rat pancreases. Glucagon (**a**, **b**), SST (**c**–**f**) and insulin (**g**, **h**) output from isolated perfused rat pancreases is shown in response to infusion with UCN3 (10 nmol/l) or SST-14 (10 nmol/l, positive control), during perfusion with 3.5 mmol/l glucose (**a**–**d**) or 10 mmol/l glucose (**e**–**h**). (**i**–**l**) Insulin output from isolated perfused rat pancreases during perfusion with 3.5 or 10 mmol/l glucose is shown in response to simultaneous infusion with CRHR2 antagonists antisauvagine-30 and K41498 (200 nmol/l) without (**i**, **j**) or with (**k**, **l**) infusion of a mixture consisting of SSTR2, -3 and -5 antagonists. Outputs are shown as min–min outputs (**a**, **c**, **e**, **g**, **i** and **k**) or as average outputs during the respective periods of the experiments (**b**, **d**, **f**, **h**, **j** and **l**) calculated over 15 consecutive points (15 min) during each period. In (**b, h **and** j**) baseline 1 covers 0–15 min, baseline 2 covers 31–50 min; in (**l**), baseline 1 covers 0–15 min, baseline 2 covers 51–65 min. Data are presented as means ± SEM, *n* = 5 or 6. *p<0.05 (one-way ANOVA followed by Tukey post hoc test [**b**, **h**, **j** and **l**] or paired *t* test [**d**, **f**]). antag., antagonists

UCN3 has been shown to both stimulate [3, 12] and inhibit [4] insulin secretion from incubated mouse/rat islets. To investigate the direct effect of UCN3 on endocrine pancreas secretion in a more physiological model, we isolated and perfused rat pancreases with 3.5 or 10 mmol/l glucose and administered UCN3 into the arterial supply at 10 nmol/l, a concentration that strongly activates rat CRHR2 (ESM Fig. 6). At 3.5 mmol/l glucose, UCN3 almost immediately reduced glucagon output to half (*p*<0.05, *n*=5, Fig. 7a,b). After infusion was terminated, output returned to pre-stimulatory levels (*p*=0.91 vs first baseline output); glucagon output was subsequently reduced by 80% by SST-14 infusion (positive control; *p*<0.05). In the same experiments, UCN3 increased SST output three- to fourfold (*p*<0.05, *n*=5, Fig. 7c,d) but did not affect insulin secretion, which was minimal at these low glucose conditions (ESM Fig. 7a,b). When perfusing rat pancreases with 10 mmol/l glucose, UCN3 doubled SST secretion (*p*<0.05, *n*=5, Fig. 7e,f). Insulin secretion was, however, unaffected by UCN3 administration (*p*=0.68, *n*=5, Fig. 7g,h), although isolated SST-14 infusion reduced secretion by ~30% (*p*<0.05). To investigate whether endogenous UCN3 affects insulin or SST secretion, we infused the CRHR2 antagonists antisauvagine-30 and K4148 into the rat pancreas perfused at 10 mmol/l glucose at a concentration that efficiently inhibits UNC3-mediated activation of CRHR2 (ESM Fig. 6, *n*=5). This increased insulin secretion approximately twofold (*p*<0.05, *n*=5, Fig. 7i,j). In the same samples, glucagon output was again reduced by UCN3 when perfusing with 3.5 mmol/l glucose but not when perfusing with 10 mmol/l glucose (ESM Fig. 7c,d). Since UCN3 increased the secretion of SST and because SST is a powerful insulinostatic regulator, we investigated SST signalling dependence for the increased insulin secretion in response to CRHR2 blockage. To this end, we infused a combination of SST receptor (SSTR) antagonists (SSRT2, -3 and -5) known to efficiently block SST signalling [13] before and during infusions of the CRHR2 antagonists. Infusion of the SSTR antagonists transiently increased insulin secretion by 30–50% (*p*<0.05, *n*=5, Fig. 7k,l) and prevented the CRHR2 antagonists from increasing insulin secretion. Secretion was thus significantly lower than during SSTR antagonist infusion (*p*<0.05). Subsequent change of the glucose in the perfusate from 10 mmol/l to 3.5 mmol/l further decreased insulin secretion by ~66% (*p*<0.05, *n*=5, Fig. 7k,l), confirming responsiveness.

## Discussion

The purpose of this study was to clarify the acute effect of UCN3 on glucose tolerance and to investigate the mechanisms involved. In our first *in vivo* study, blood glucose concentrations in rats receiving glucose orally and either saline (control group) or UCN3 subcutaneously were similar, apparently indicating that UCN3 did not affect intestinal glucose entry, absorption, or disposal. However, this was clearly not the case since both gastric emptying and glucose absorption and insulin secretion were reduced by >50%, in agreement with previous findings in mice and rats [14, 15]. Here, we show that the maximal efficacy of UCN3 was comparable with that of GLP-1 but that the effect was more sustained, possibly because of a longer t½ of UCN3. Indeed, we found that the t½ of UCN3 was about 2 min after i.v. injection, whereas others reported the t½ of GLP-1 in rats to be ~0.8–1 min (i.v.) [16, 17]. Glucose absorption depends on gastric emptying, intestinal motility, and intestinal blood flow, and UCN3 may to some extent compensate for the delayed gastric emptying by increasing intestinal blood flow through vasodilatation [18]. Our finding of *Crhr2* in the gastrointestinal arterioles supports this hypothesis, although in our study, glucose absorption after UCN3 was still reduced to one-third as judged by plasma 3-OMG concentrations. Despite its short-lasting effect on gastric emptying, GLP-1’s reduction of glucose absorption was even more pronounced than that of UCN3, possibly because of additional inhibitory effects on intestinal motility [19]. Given that UCN3 concentrations are high in the gut, and because CRHR2 appears to be expressed by L cells [20], we hypothesised that the effects of UCN3 on gastric emptying, and potentially also on blood glucose, could be mediated indirectly by UCN3-regulated secretion of one or more of the glucoregulatory gastrointestinal hormones. However, UCN3 clearly did not affect GIP, GLP-1, NT or SST secretion from isolated perfused rat small intestine and presumably also would not have affected peptide YY secretion since peptide YY is co-secreted with GLP-1 [21]. Instead, UCN3’s restrictive effects on gastric emptying may be mediated through direct effects on the pacemaker activity in the stomach and/or via the enteric nervous system since we found *Crhr2* expression in the gastric submucous plexus and in the submucosal surface of the circular smooth muscle layer of the pyloric antrum.

With regards to UNC3’s effects on blood glucose, previous studies in rats and mice, using 90–100 nmol/kg UCN3, have shown reduced glucose tolerance following an OGTT [3–5]; conversely, genetic *Ucn3* ablation in mice or antagonism of CRHR2 in rats led to improved glucose tolerance [4, 5]. It was therefore puzzling why our first in vivo study showed UCN3 to have no effect on post-challenge blood glucose excursions. One possibility is that the inhibitory actions of UCN3 on glucose absorption at the applied dose cancelled out the concomitant increase in blood glucose resulting from the UCN3-induced inhibition of insulin secretion. To investigate this possibility, we subsequently compared the effects of 2, 7.5 and 30 nmol/kg again with GLP-1 as a reference point. The low UCN3 dose reproduced the findings of the first in vivo study: no net effect on post-challenge blood glucose levels despite pronounced inhibition of gastric emptying. In agreement with previous studies [3, 4], the two higher concentrations resulted in glucose intolerance. These results point to differential organ sensitivity towards UCN3 since full efficacy on inhibition of gastric emptying was reached before full efficacy on insulin secretion. This observation raises the question of the magnitude and regulation of the physiological plasma concentrations of UCN3. For UCN3 to physiologically affect gastric emptying and postprandial glucose absorption and assimilation, the secretion of UCN3 should increase in response to glucose intake, an expectation that remains uninvestigated. We attempted to quantify UCN3 in venous effluents from isolated perfused rat small intestine preparations stimulated with luminal glucose (20% wt/vol.) but, despite several attempts, we were unable to find a sufficiently sensitive and specific UCN3 assay.

With regards to the mechanisms underlying UCN3-mediated suppression of insulin secretion, our expression data on rat islets suggested that this may occur indirectly through SST since *Ucn3* was highly abundant in beta cells but not in alpha cells or delta cells, while *Crhr2* was exclusively detected in delta cells. These expression patterns are consistent with previous findings [4, 22, 23]. In human islets, expression patterns were similar with the only difference being that *UCN3* was also expressed in alpha cells, consistent with previous reports [4, 23, 24]. However, the physiological role of UCN3 expression in alpha cells has not been clarified and the role of UCN3 in the beta cell remains debated. An early study showed that UCN3 stimulated rather than inhibited insulin secretion from rat islets [3]. Consistent with the expression pattern, UCN3 stimulated SST secretion from isolated perfused rat pancreases perfused at 10 mmol/l glucose, in line with data from incubated or perifused mouse islets [4]. However, in contrast to these data [4], increased SST secretion did not translate into detectable inhibition of insulin in our study. The reason for this difference may be our use of an intact pancreas as opposed to dispersed islets. Alternatively, the UCN3 dose we used (10 nmol/l) may have been insufficient to activate CRHR2 further. Although intra-islet UCN3 concentrations are unknown, concentrations may be in the nanomole range as it is highly expressed by beta cells and is co-secreted with insulin, possibly resulting in an intra-islet concentration of 1 μmol/l [25] during high glucose conditions. In any case, our perfusion studies with CRHR2 antagonists suggest an important inhibitory role for UCN3 in insulin secretion, since output doubled in response to the antagonists, consistent with the data from perifused islets demonstrating that insulin secretion (at 16.8 mmol/l glucose) from *Ucn3-*null mice was two- to threefold higher than the insulin secretion from wild-type littermates [4]. Furthermore, we demonstrated that the response could depend on SST secretion since it was abolished by combined blockage of SSTR2, -3 and -5, the major SSTRs expressed by alpha cells and beta cells [26], which again is consistent with reported data [4]. Less is known about UNC3’s regulatory role during low glucose conditions. During perfusion at 3.5 mmol/l glucose, UCN3 stimulated SST secretion and inhibited glucagon secretion, This finding contrasts to those of previous reports involving incubated rat islets [3, 5] but aligns with the finding in our and other studies that CRHR2 is exclusively detected in delta cells [4, 23, 24]. Besides the anticipated differences in intra-islet UCN3 concentrations at high vs low glucose concentrations, the difference we found in glucagon and insulin responses to UCN3 administration is presumably also influenced by glucagon being more sensitive than insulin to paracrine inhibition by SST [26].

In this study, we did not observe any changes in the plasma concentrations of glucagon in vivo following UCN3 administration, in contrast to previous reports also arising from rat studies [5]. The explanation is likely to be that glucagon secretion was already robustly inhibited at baseline conditions in the in vivo study where blood glucose was ~6 mmol/l. Alternatively, any inhibitory effect of UCN3 on glucagon secretion in vivo may have been balanced by UCN3-mediated secretion of corticosterone and catecholamines (adrenaline [epinephrine] and noradrenaline [norepinephrine]), since these hormones increase glucagon secretion [27–29]; this warrants further investigation.

### Conclusion

Our study highlights UCN3 as being a powerful inhibitor of gastric emptying and shows that it delays glucose absorption by more than 50%. At the same time, UCN3 inhibits glucose-stimulated insulin secretion by paracrine mechanisms that depend on SST secretion. As such, UCN3 may act as a so-called ‘decretin’ [30], a hormone that opposes the incretin hormones and thus suppresses glucose-stimulated insulin secretion. However, the net effect of UCN3 on postprandial blood glucose will ultimately be determined by circulating concentrations of UCN3, since our study show that the inhibition of gastric emptying is more UCN3 sensitive than the inhibition of insulin secretion. Unfortunately, we were unable to measure UCN3 in rat plasma or in perfusates from isolated perfused rat small intestine. Further studies are thus needed to determine whether UCN3 is in fact a hormone in the classical sense and whether it acts as a decretin in a physiological setting; this would at least require that UCN3 secretion is stimulated by glucose and that the increase precedes the blunted insulin secretion.

## Supplementary Information

ESM 1 (PDF 407 kb)

## Data Availability

This study does not involve generation of sequencing data. Raw data can be obtained upon reasonable request to the corresponding author.

## Abbreviations

3-OMG: 3-*O*-Methyl-d-glucose
CRHR2: Corticotropin-releasing hormone receptor 2
GIP: Glucose-dependent insulinotropic polypeptide
GLP-1: Glucagon-like peptide-1
NT: Neurotensin
SST: Somatostatin
SSTR: SST receptor
UCN3: Urocortin-3
