# Supplementary material for: Opposing roles of the entero-pancreatic hormone urocortin-3 in glucose metabolism in rats
Source: Diabetologia. 2022 Mar 24;65(6):1018–31. doi: 10.1007/s00125-022-05675-9 (PMC9076751; doi:10.1007/s00125-022-05675-9)
Supplement: Supplementary file 1 — (PDF 407 kb) [file 125_2022_5675_MOESM1_ESM.pdf]

Electronic supplementary material

The opposing roles of the entero-pancreatic hormone, urocortin-3, in glucose metabolism in rats

Kaare V. Grunddal[1], Samuel A. J. Trammell[2], Cecilie Bæch-Laursen[1,2], Daniel B. Andersen[1,2], Stella F.S. Xu[1,2], Helle Andersen[3], Matthew P. Gillum[2], Seyed M. Ghiasi[4,5], Ivana Novak[4], Björn Tyrberg[6,7], Chien Li[8], Mette M. Rosenkilde[1], Bolette Hartmann[1,2], Jens J. Holst[1,2,*], Rune E. Kuhre[1,3,*]

Supplementary methods

Animals and ethical considerations. Animal studies were conducted with permission from the Danish Animal Experiments Inspectorate (2018-15-0201-01397 and 2020-15-0201-00756) and from the local ethical committee in accordance with the guidelines of Danish legislation governing animal experimentation (1987) and the National Institutes of Health (publication number 85-23). Male Wistar rats (~250g) were obtained from Janvier (Saint Berthevin Cedex, France) and housed with ad libitum access to standard chow and water, following a 12:12 h light:dark cycle. Rats was acclimatized for a least a week before experiments.

Chemicals and peptides: UCN3 (mouse) trifluoroacetate salt and GLP-1 7-36amide trifluoroacetate salt were from Bachem (cat. no. 4040194 and 4030663, Bubendorf, Switzerland). Antisauvagine-30and K41498 (CRHR2 antagonists) were from Tocris (cat. no. 2071, 2391, and 2070 (Abingdon, UK). D-glucose, L-arginine monohydrocholoride, acetaminophenand 3-Isobutyl-1-methylxanthine Cat. no. I5879) (IBMX) were from Sigma Aldrich (Cat. no. G8270, A5131, A7085, and I5879, Brøndby, Denmark).

UCN3 half-life in rats: The half-life of subcutaneous UCN3 was determined using samples from the *in vivo* study. For the intravenous half-life study, rats were housed and anesthetized as described above. After confirmation of surgical anesthesia, rats were opened by a midline incision and a needle was inserted into the abdominal vena cava inferior (for injection of UCN3) and another was inserted into the portal vein (for collection of blood samples). Baseline samples were collected at -5 and 0 min. Immediately after collection of the 0 min sample, UCN3 was injected (2 nmol/kg; prepared in isotonic saline + 0.1% (w/v) HSA so injection volume was ~ 200µL) through

the catheter in the abdominal vena cava inferior, and blood was collected at time 1, 2, 5, 10, 15, 20, and 30 min (200 µL/time point). Samples were immediately transferred onto ice and centrifuged (2400 g, 15 min, 4°C) within 30 min after collection. Plasma was transferred into fresh eppendorf tubes and stored at -20°C.

Perfusion studies: Rats were housed and anesthetized as described above and weighed ~ 300 g at the day of experiment. The pancreas or the small intestine was isolated and perfused in situ as described in detail elsewhere1 2. For both, we used a UP100 Universal Perfusion System from Hugo Sachs (Harvard Apparatus, March-Hugstetten, Germany) which heated the perfusion buffer to 37°C and allowed continuous recording of perfusion pressure. A modified Krebs–Ringer bicarbonate buffer, that had been added 0.1% (w/v) BSA (faction V; cat. no. 1.12018.0500), dextran T70 (to balance osmolarity; Pharmacosmos, Holbaek, Denmark), and with 5 mmol/l pyruvate, fumarate, and glutamate was used for perfusion. Furthermore, perfusion buffer was supplemented with 10 µM 3-isobutyl-1.methylxanthine (IBMX, cat. no. I5879, Sigma Aldrich, Brøndby, Denmark) for intestinal perfusions. The buffer was constantly gassed with 95% O2/5% CO2 to maximally increase pO2 and maintain pH around 7.4. Methods are described in more details elsewhere[3, 4]. For small intestine experiments, the large intestine was removed after tying off the supplying vasculature. A plastic tube was inserted into the proximal part of the small intestine, just distal to the part of the duodenum associated with the pancreas, and the gut was emptied for its contents by careful flushing with isotonic saline (room temperature). Next, a steady flow of saline (0.5 mL/min) was applied through the lumen. A catheter was inserted into the upper mesenteric artery for arterial perfusion (7.5 mL/min), another into the portal vein for venous effluent collection. Once the catheters were secured, the rat was killed by diaphragm perforation and the preparation was left to equilibrate for 30 min before collection of the first sample. Effluent samples were collected in one-minute intervals and instantly transferred to ice. After collection of baseline samples (first 15 min), the gut was stimulated with luminal glucose (20% w/v) prepared in isotonic saline) at 2.5 mL/min for the first 5 min (to quickly replace the saline in the lumen) and at 0.5 mL/min for the reaming stimulation period. Hereafter, a similar bolus of isotonic saline was administered for the first 5 min (to quickly replace the glucose solution) followed by 0.5 mL/min the remainder of the experiment. Glucose concentrations in venous effluents were quantified immediately by use of a handheld

glucometer as specified in the biochemical measurements section. In same experiments, the gut was stimulated with intra-arterial glucose at a concentration which for each experiment matched the highest measured glucose concentration measured in the venous effluents following luminal glucose administration. Glucose was prepared in perfusion buffer (0.5 M) and was administered through a three-wave stop-cock at rates <0.2 mL/min. At the end of the protocol, 0.1 mmol/l taurodeoxycholic acid was administered intra-arterially to confirm responsiveness (pos.control). For pancreas perfusions, the pancreas was isolated by removing the stomach, spleen and small and large intestine (except for the part of duodenum connected to the pancreas) and by tying off the renal stalks. The abdominal aorta was tied off just below the diaphragm and, immediately after, a catheter was inserted retrogradely into the abdominal aorta just distally to the renal arteries so that the aorta exclusively supplied the pancreas (through both the coeliac and the superior mesenteric arteries). A perfusion flow of 5.0 mL/min was used. As was the case for small intestine perfusion, venous effluent was collected through a catheter in the portal vein and effluents were collected each minute. Test stimuli were administered via the aorta through a three-way stop-cock, were prepared in perfusion buffer without use of solubilizers, and included glucose, UCN3 (final concentration = 10 nM) or antisauvagine-30+K41498 (final concentrations = 200 nM). For both small intestine and pancreas perfusions, perfusion pressure and perfusion output was monitored closely during the experiments and experiments were disregarded (less than 15%) if these parameters varied more than 10% during the course of the experiment.

Pharmacology studies: COS-7 cells, that were originally purchased from ATCC (VA, USA, Cat# CRL-1651) and were tested negative for mycoplasma infection, were grown in DMEM supplemented with 2 mmol/l L-glutamine, 180 U/ml penicillin, 45 µg/ml streptomycin, and 10 % (w/v) FBS according to protocols described previously[1]. 35,000 cells were seeded per well into 96-wll plates coated with poly-D-lysine and modified for either mouse or rat CRHR2 expression using transient calcium phosphate precipitation transfection procedure[2], using pCMV6-Entry vectors containing untagged Crhr2 clones for either the rat or mouse version of the receptor (Cat. no. RN206899 and MC208334, OriGene Europe, Herford, Germany). On the assay day, two days after transfection, cell medium was replaced with HBS buffer containing 1 mmol/l IBMX and cells were left to equilibrate for 30 min at 37°C. Cells were stimulated for 30 min at 37°C with synthetic

mouse UCN3 at a concentration range of $1x10^{-12}$ to 1×10^{-7} M (using 10-fold stepwise increases from lowest to highest concentration). Stimulations were performed in duplicates. After stimulation, cells were washed once in HBS buffer and intra-cellular cAMP concentrations were quantified by use of *in vitro* HitHunter cAMP assay (based on enzyme fragment complementation, DiscoverRx) carried out according to the manufacturer's instructions. Data are expressed relative to the response at 10^{-7} M (UCN3). In another line of experiments, inhibitory properties of three described CRHR2 antagonists (Antisauvagine-30and K41498) were tested. The protocol outlined above was used but instead of stimulating with different UCN3 concentrations, cells were exposed to either Antisauvagine-30 andK41498 which were added in concentration rages from $1x10^{-12}$ to 1×10^{-7} M (10 fold increases) in the presence or absence of 10 nM UCN3. The experiments were done three times independently.

UCN3 concentrations in rat gut and pancreas: UCN3 concentrations in rat gut and pancreas: Gastrointestinal and pancreas tissue biopsies (~1-1.5 cm/piece) were collected from rats that had been housed and anesthetized as described above. Tissue was thoroughly rinsed in PBS and immediately after snap frozen on dry ice before storage at -80°C. Tissue samples were homogenized in 1% trifluoroacetic acid (TFA) (Cat. no. TS-28904, Thermo Fischer Scientific, MA, USA) with a 5 mm steal bead and bead mill (TissueLyzer, Qiagen instriments AT, Hombrechtikon, Switzerland). Total protein concentrations were quantified by Pierce BCA Protein Assay Kit (Cat. no. 23227, Thermo Fisher Scientific), extracts were purified using tc18 cartridges (Cat. no. 036810 Waters, Massachusetts, USA), and samples were reconstituted in 1 mL 80 mM phosphate buffer containing 0.1% human serum albumin, 10 mM EDTA and 0.6 mM thimerosal. UCN3 concentrations were quantified as described in the biochemical measurements section and were expressed normalized to total protein.

Expression analysis of UCN3 and CRHR2 in rat and human islets: Ucn3 and *Crhr2* expression was investigated in rat islets by qPCR and in human islets by re-analyzing publicly human islet single-cell sequencing data from donors with type 2 diabetes and non-diabetic donors (n=4 with diabetes, n=6 without diabetes) were used for this analysis [16]. Expressions were compared to more well-known islets genes; proglucagon (*Gcg*), *Glp-1r* and *Ins-1 and Ins-2* in case of rat islets and to *GCG, UCN1, UCN2, and GLP-1R* in human alpha-, beta- and delta-cells.

Dual in situ hybridization and immunofluorescence:

Male Wistar rats (~ 300 g) were anaesthetized by a subcutaneous injection of Hypnorm/Midazolam as described above. Organs were excised while the rats were alive after tying off the respective supplying vasculature, and included pancreas, gastric ventricle, jejunum, distal ileum, mid colon and thigh muscle. Tissue samples were immediately transferred into fresh 4% (w/v) paraformaldehyde, PBS, and fixed for 24h at room temperature. Tissues were then dehydrated, paraffin-infiltrated (Shandon Excelsior, Thermo Fisher) and embedded in paraffin blocks. Sections of 4 µm were cut using a microtome (Cat. no. HM355S, Leica) and mounted on slides (Superfrost Plus Slides, Thermo Scientific). The slides were heated at 60°C for 1h, dewaxed and rehydrated using xylene and graded dilutions of ethanol. The *in situ* hybridization was performed using custom designed mRNA specific probes (listed in supplementary table 1) in accordance with supplied manual from the manufacturer (RNAscope 2.5 HD detection kit, Cat.no. 322360, Advanced Cell Diagnostics, Milan, Italy) after which sections were counterstained with hematoxylin and coverslipped or further stained by means of immunofluorescence. After the *in situ* hybridization, slides were blocked in blocking buffer (2% (w/v) BSA prepared in PBS for 10 min) and incubated with primary insulin, glucagon or somatostatin antibody in blocking buffer) over night at 4°C. The following day, slides were washed and incubated with Alexafluor 488 conjugated secondary antibodies in blocking buffer for 1 h at room temperature (Antibodies are listed in supplementary table 1). Coverslips were then mounted with ProLong Gold 32 Antifade Mountant with DAPI (Cat. no. P-3693 I, Thermofisher). The slides were analyzed using a Zeiss Widefield fluorescence microscope and AxioCam 506 mono camera or a Zeiss Axio Scan.Z1 Slide Scanner and AxioCam MRm camera. Control studies revealed no unspecific binding of the secondary antibodies.

Biochemical measurements: In vivo study: Blood glucose was measured by a handheld glucometer (Accu-check Compact plus device; Roche, Mannheim, Germany) (study 1) or by use of a semi-automated blood glucose analyzer (Biosen, C-line, EKF Diagnostics, Cardiff, UK) (study 2). Plasma acetaminophen were measured by an spectrophotometry assay (Cat. no. 506-30, Sekisui Diagnonstics, Pfugstadt, Germany) (study 1) or on a semi-

automated cobas 6000 analyzer (Roche, Basel, Switzerland) (Cat. no. 06769942190) (study 2). Furthermore, FFA, glycerol and TAG was measured on the same cobas analyzer (Cat. no. E434-91795, F6428, and 20767107322). Plasma glucagon (intact, 1-29) and plasma insulin was quantified by ELISAs from Mercodia (Cat. no. 10-1250-01 and 10-1281-01, respectively, Uppsala, Sweden). UCN3 in rat plasma (UCN3 half-life studies) were quantified by ELISA (Cat. no. YK200, Eagle Biosciences, NH, USA). Samples for 3-OMG quantification was prepared and quantified as following: *Sample Preparation:* Unless otherwise stated, all chemicals used below were provided by Merck at either liquid chromatography-mass spectrometry (LCMS) grade or highest purity. Rat plasma from the *in vivo* study was thawed on wet ice prior to extraction. 10 µL of rat plasma contained in a 1.5 mL centrifuge tube was dosed with 10 µL of internal standard (0.3 mmol/l $^{13}C_6$-3-o-methyl-glucose ($^{13}C_6$-3-OMG), chemical purity: 100.0%, isotope enrichment: 99 atom-%, Omicron Biochemicals, Inc., Indiana, USA, catalog #: GLC-120)). 180 µL of LCMS grade acetonitrile was added to each sample. Immediately after addition, the sample was vortexed briefly (~3s, top speed) and repositioned in the wet ice. Next, samples were placed into a pre-heated (80°C) Eppendorf Thermomixer and shaken at top speed (1,400 RPM) for 5 min. Samples were placed in wet ice for 5 min and were then centrifuged (20,238 x *g*, 5 min at room temperature). Clarified supernatants were transferred to fresh 1.5 mL centrifuge tubes, and tubes were dried via speed vacuum for ~1.5 h. Quality control calibrants and standard curve calibrants were prepared from separate aliquots of pure, non-labeled 3-OMG. Quality controls (QCs) were composed either in the presence of plasma from a naïve rat (10 µL) or in the presence of LCMS grade water (10 µL) in triplicate at each concentration level. In both cases, 10 µL of either 140, 1,400, or 2,800 µM solutions were combined with internal standard (same concentration and volume as above). Any QC containing rat plasma was extracted with 270 µL of LCMS grade acetonitrile. An additional rat plasma aliquot (10 µL) was adulterated with 10 µL of LCMS water to serve as a background blank. These QCs were then extracted as above. All non-plasma containing QCs were extracted in a similar manner to above, except that heating was omitted. A calibration curve was composed by adding 10 µL of standard (either 0, 7, 17.5, 26.25, 35, 70, 175, 262.5, 350, 700, 1,750, 2,625, or 3,500 µM) to internal standard (same concentration and volume as above). Extraction was carried out in the same manner as non-plasma containing QCs. A blank

extraction containing only LCMS grade water (30 µL) and LCMS grade acetonitrile (270 µL) was extracted in the same manner as non-plasma containing QCs and standards. After drying, all samples, QCs, and standards were re-suspended in 100 µL of 95/5% (v/v) LCMS acetonitrile/water. Any plasma containing QC or sample was centrifuged as above. The clarified supernatants were transferred to fresh autosampler vials (Verex vials, catalog AR0-3920-12, Phenomenex ApS) and capped (Verex Cert+MSQ PTFE cap (catalog AR0-8952-12-M, Phenomenex ApS)). All were placed in a 6 °C autosampler to await analysis. Samples were injected (10 µL) in a random order. QCs were injected at the beginning, middle, and end of sample analysis. The calibration curve was performed once all samples had been analyzed. The first round of QC injections was repeated after the calibration curve.

LC-MS based Quantification of 3-OMG: Prior to beginning analysis, the instrument was calibrated by directly infusing sodium acetate. Calibration was modeled with HPC mode. Calibrant was also infused at the beginning of each analysis. A lock mass (Hexakis [1H, 1H, 2H-perfluoroethoxy] phosphazene in 2-propanol [0.1 mg/ml]) was employed to improve mass accuracy over the duration of analysis (*m/z*: 680.0355). Analytes were separated using a Phenomenex amide column (2.1 x 100 mm, insert) with a gradient between mobile phase A (0.006% (v/v) ammonium hydroxide in LCMS grade acetonitrile) and B (5 mmol/l ammonium acetate + 0.006% (v/v) ammonium hydroxide in LCMS water) with a flow rate of 0.4 mL/min. The gradient was as follows: initial: 5% B, 0 – 5 min: 100% B, 5 – 10 min: 100% B, 10.1 – 15 min: 5% B. The column was maintained at 25 °C. Analytes ionized via electrospray were detected using a Bruker Impact II Q-TOF operated in full scan (*m/z*: 50 – 1000 Da), negative ion mode. The instrument was operated using the following conditions: cone voltage, 3 kV; collision energy, 7 eV; nebulizer gas flow, 10 L/min; nebulizer gas temperature, 220°C; nebulizer pressure, 2 bar; and scan rate, 2 Hz. 3-OMG was detected using its intact *m/z* ratio 193.0717 (±0.005 Da) as well as its in-source fragment ($[M\text{-}CH_4O]^-$ = 161.0456 (±0.005 Da)). The ions eluted at 2.79 ± 0.5 min. Quantification was based upon its in-source fragment. Molar amounts were determined using a weighted (1/X) linear standard curve, whereby the concentration versus the ratio of the external standard to the internal standard was plotted.

ESM, table 1: Antibody tables for *in situ* hybridization studies.

Target mRNA	Target species	Manufacturer/ Provider and Cat. no.
Ucn3	Rattus norvegicus	ACD biotechne(Newark, CA), 580691
Crhr2	Rattus norvegicus	ACD biotechne, (Newark, CA), 417851
Ppib	Rattus norvegicus	ACD biotechne, (Newark, CA), 313921
DapB	Bacillus subtilis strain SMY	ACD biotechne, (Newark, CA), 310043

ESM, table 2: Probe tables for *in situ* hybridization studies

Target	Manufacturer/ Provider and Cat. no.	Host	IHC dilutions for *in situ* hybridization	IHC dilutions for secretin localization
Insulin	In-house, 2006-4	Guinea Pig	1:3000	-
Somatostatin	In-house, 1759	Rabbit	1:3000	-
Glucagon	Abnova (Taipei, Taiwan) MAB1500	Mouse	1:300	-
CF488A anti-Guinea pig IgG	Biotium (Fremont, CA), 20169	Donkey	1:200	-
Alexa fluor 488 anti-rabbit IgG	Abcam (Cambridge, UK), ab150109	Donkey	1:200	-
Alexa fluor 488 anti-rabbit IgG	Abcam (Cambridge, UK), ab150081	Goat	1:200	-
Biotinylated anti-rabbit IgG	Vector Laboratories (Burlingame, CA), BA-1000	Goat	-	1:200

ESM, table 3. Primer sequences for qPCR-analysis of rat islets.

Primers

Gene	Forward	Reverse
Crhr2	CTGGAACCTCATCACCACCT	AGCCTTCCACAAACATCCAG
Gcg	AACAACATTGCCAAACGTCA	CAGCTATGGCGACTTCTTCC
Ucn3	CTTCAGCTGCCTCAACACAG	GCTGTGCTTGGGATTGGTAT
Glp-1r	ATCCACCTGAACCTGTTTGC	GCAGTATTGCATGAGCAGGA
Ins-1	GGGGAACGTGGTTTCTTCTAC	CCAGTTGGTAGAGGGAGCAG
Ins-2	CAGCACCTTTGTGGTTCTCA	CACCTCCAGTGCCAAGGT
Gapdh	TGACAACTTTGGCATCGTGG	GGGCCATCCACAGTCTTCTG

ESM, table 4. Assay information's, quantification of gut and pancreas hormones.

Assay	Antibody code	Epitope	Sensitivity (pM)	Sepecificity	Linear range (pM)
GIP (total)	80867-5	C-terminal	1	Reacts fully with intact and truncated GIP	1-160
Glucagon total	4305	C-terminal	1	Detects all bioactive forms	1-200
GLP-1 (total)	390	Amidated C-terminal	1	Reacts fully with intact and truncated GLP-1	1-200
Insulin	2006-3		1	Detects all bioactive forms	1-160
Neurotensin (total)	3D97	N-terminal	1	Detects all bioactive forms	1-200
Somatostatin	1758-5	Side-viewing	1	Detects all bioactive forms	1-100

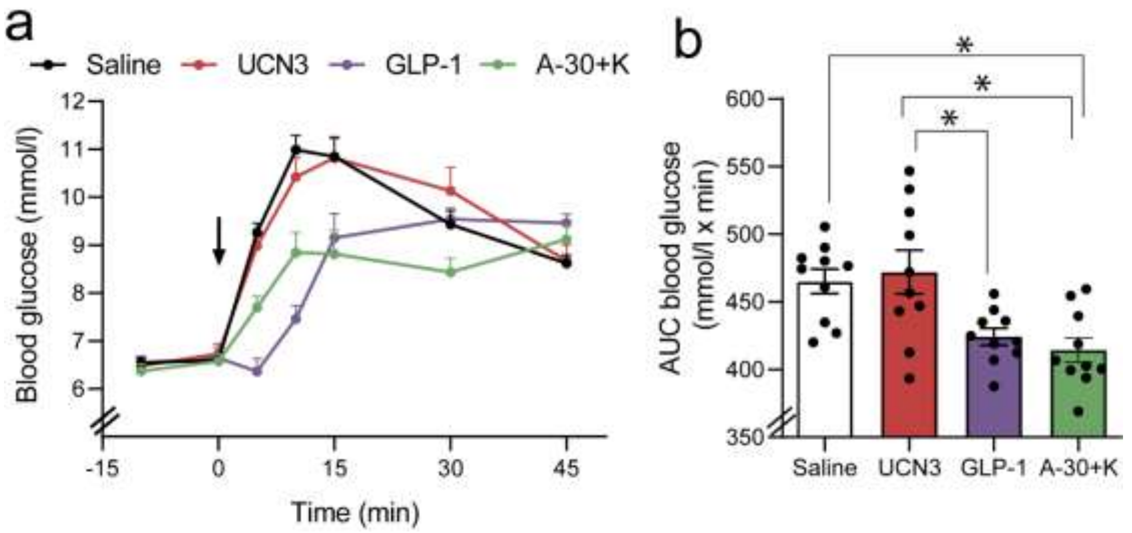

ESM, figure 1. Effects of activation and blockage of CRHR2 signaling on blood post-challenge glucose regulation in rats. Data are shown as means+SEM in response an OGTT (2 g/kg) combined with subcutaneous injection of saline (black), UCN3 (30 nmol/kg, red), GLP-1 (30 nmol/kg, purple), and the two CRHR2 antagonists Antisauvagine-30+K41498 (100 µg/kg). a: time dependent effects of treatments. Arrow indicate time of OGTT and subcutaneous injection. b: AUCs (calculated from time -10 to 45 min). Baseline samples were collected at time -10 and -5 min and are plotted as -10 and 0 min. *p<0.05, n=10. Data from saline, UCN3 and GLP-1 group is also presented fig. 1a,b).

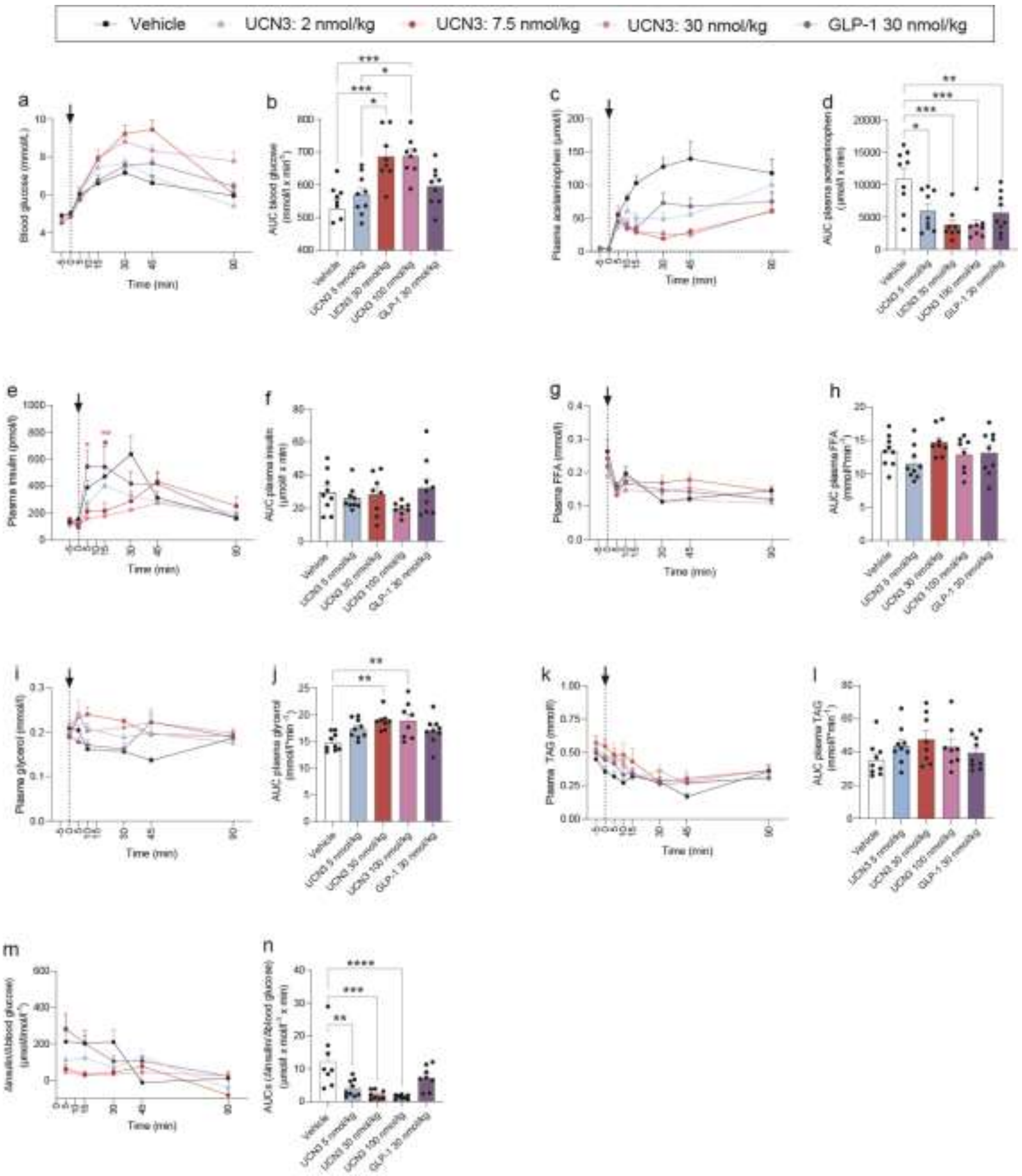

ESM, figure 2. Dose-dependent effects of UCN3 on blood glucose, gastric emptying, glucose-stimulated secretion of insulin and lipolysis in the rat. Data are shown as means+SEM in response to: a-p: an oral glucose (2 g/kg) challenge in combination with s.c. injection of either vehicle (control), three different doses of UCN3 (2, 7.5 and 30 nmol/kg), or GLP-1 (7-36amide, 30 nmol/kg). a, b: blood glucose, c,d: plasma acetaminophen, e,f: plasma insulin, g, h: Δinsulin/Δblood glucose (pmol/l x min^{-1}/mmol/l x min^{-1}), i,j: plasma FFA, k,l: plasma TAG, and m, n: plasma glycerol. AUCs in b, d, f, j, l, and n covered -5 to 90 min, whereas AUCs in h was from 5 to 90 min. Baseline sample points taken at -30 and -15 min were in alle cases plotted as -5 and 0 min). **p*<0.05, ***p*<0.01, ****p*<0.001. n=8-9. Abbreviations: Free fatty acids: FFA and Triacylglycerol: TAG. Data are a representation of data in fig.2, but in this case also including the 90 min point.

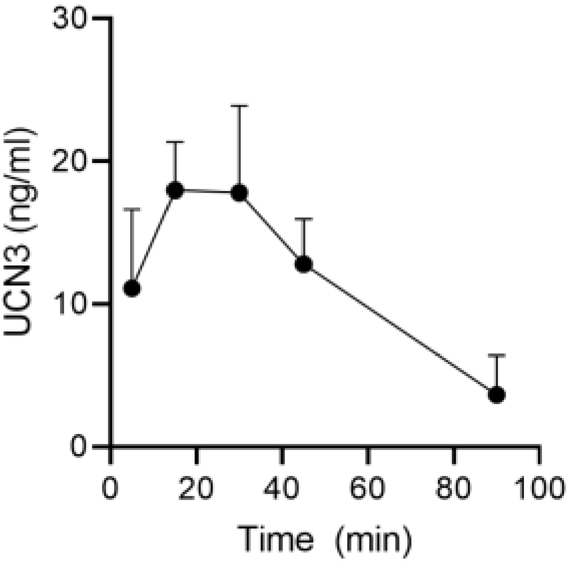

ESM, figure 3: Plasma concentrations of UCN3 after subcutaneous administration of UCN3 at a dose of 30 nmol/kg. Data are shown as means±SEM. n=8. Plasma samples are from the in vivo experiment presented in figure 2.

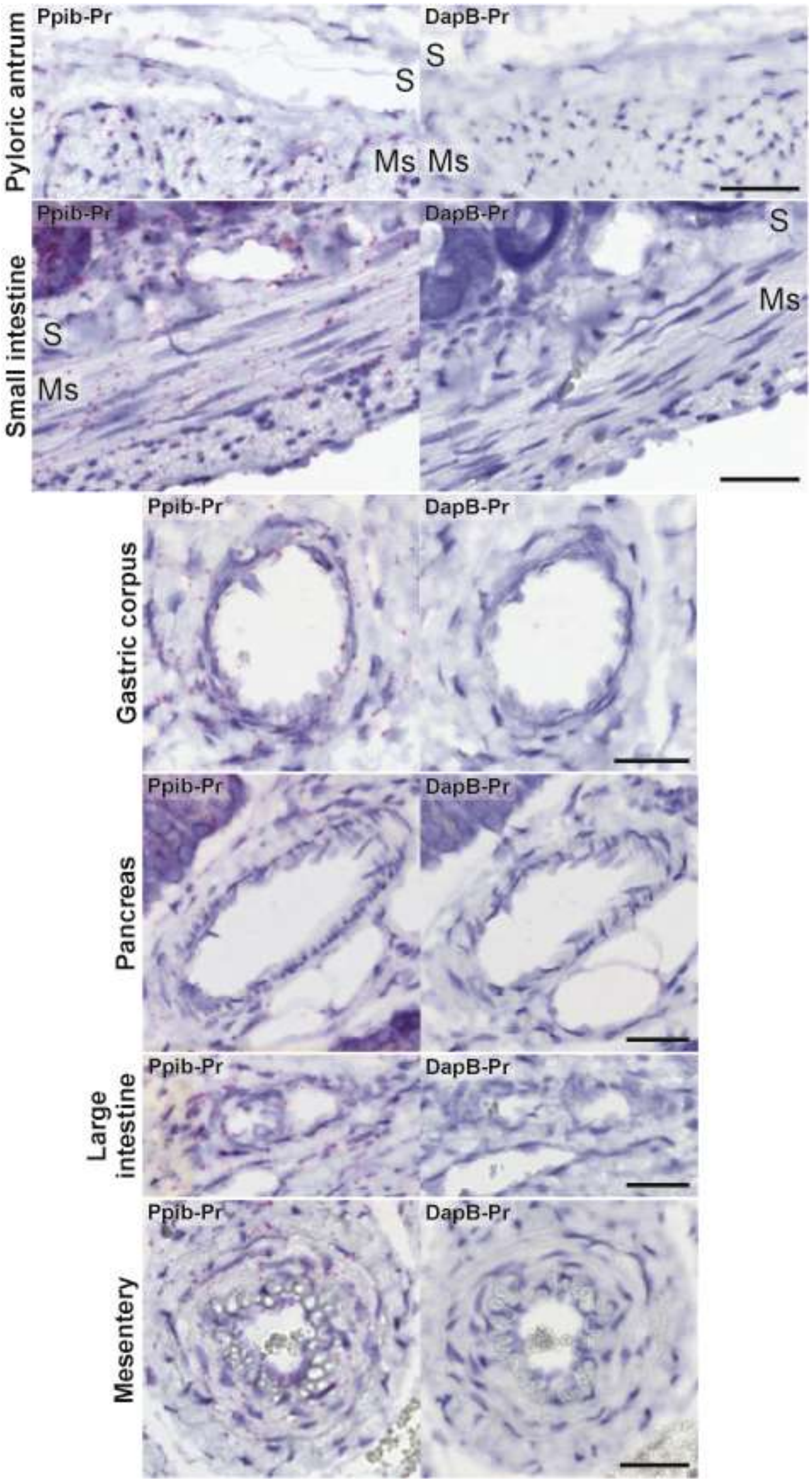

ESM, figure 4. Chromogenic ISH control studies of rat GI tract. Representative images of gastrointestinal submucosa stained with positive control probe targeting Rattus norvegicus Peptidylpropyl isomerase B mRNA (*Ppib*)or negative control probe targeting Bacillus subtilis dihydrodipicolinate reductase mRNA (*DapB*) mRNA. Red dots, single mRNA. Nuclei counterstained with hematoxylin. Bar, 30 µm. N=3 male rats.

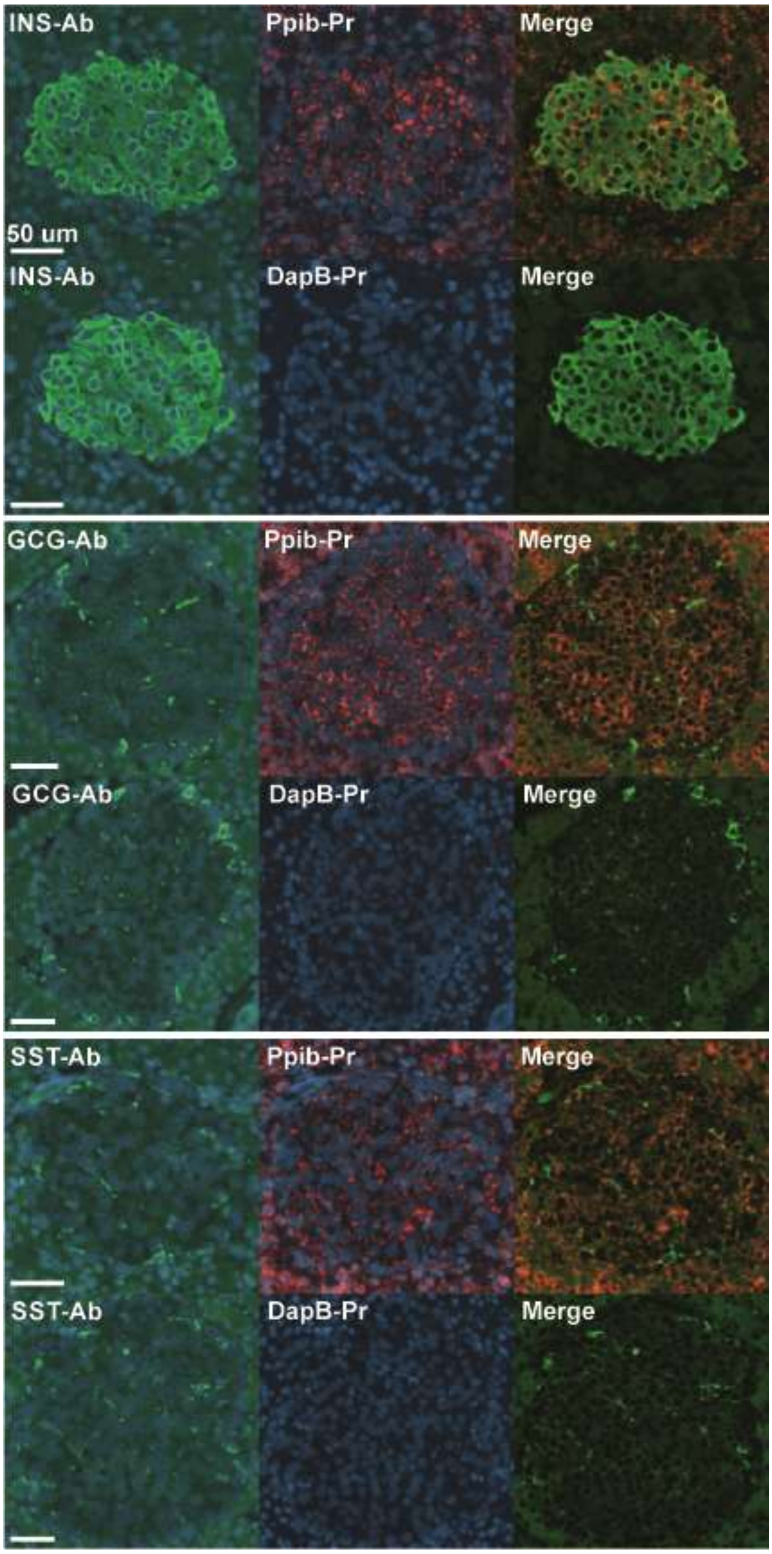

ESM, figure 5. Dual ISH and immunofluorescence control studies in rat pancreas. Representative images of pancreatic islets immunofluorescence stained for insulin, glucagon and somatostatin (green) ISH co-stained with positive control probe targeting Rattus norvegicus Peptidylpropyl isomerase B (Ppib) mRNA or negative control probe targeting Bacillus subtilis dihydrodipicolinate reductase (DapB) mRNA (red). Nuclei counterstained with DAPI (blue). Bar, 50 µm. n=3 male rats.

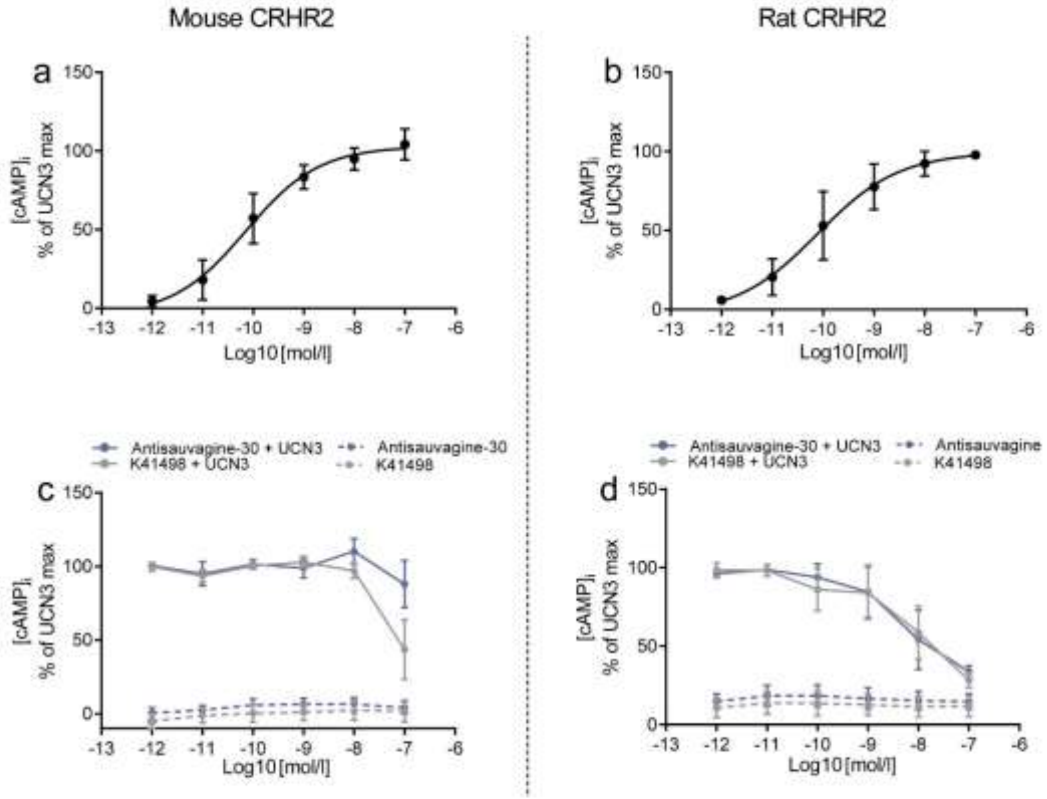

ESM, figure 6. Activation of mouse and rat CRHR2 by mouse UCN3 and antagonistic effects of antisauvagine-30 and K41498 on CRHR2. Data are shown as means±SEM. Experiments were performed on COS-7 cells transiently transfected with either a mouse or rat CRHR2 containing vector. a: Dose-response of mouse UCN3 on mouse CRHR2 activation (mirrored by $[cAMP]_i$ production), b: Dose-response of mouse UCN3 on rat CRHR2 activation, C and D: Antagonistic effects of Antisauvagine-30, and K41498 on UCN3 induced (10 nM) activation of (c) mouse or (d) rat CRHR2. The antagonists were also added alone (in the absence of UCN3) to determine any intrinsic activity (stippled lines). n=3.

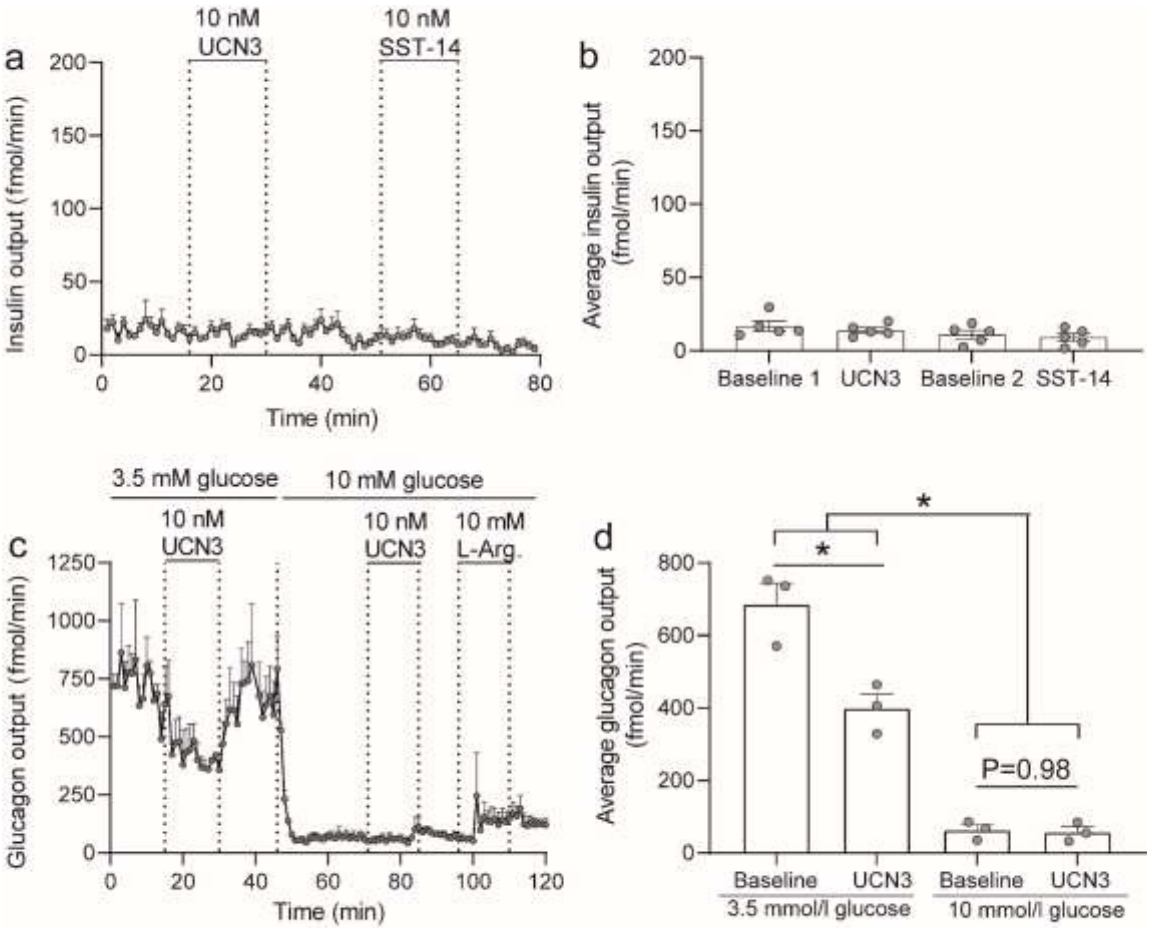

ESM, figure 7. Effects of UCN3 infusion and blockage of CRHR2 signaling on secretion of glucagon from isolated perfused rat pancreases under high glucose concentrations. Data are shown as means±SEM. a, b: insulin output at low glucose (3.5 mmol/l) in response to infusion with UCN3 (10 nmol/l) or SST-14 (10 nmol/l). c, d: Glucagon output (fmol/min) in response to infusion with UCN3 (10 nmol/l) at low (3.5 mmol/l) and high (10 mmol/l) glucose conditions. Data in a,b are the same samples as shown in figure 6a, b (in this case showing glucagon output). n (a,b) = 5, n (c, d) = 3. *P<0.05.

References

[1] Rosenkilde MM, Andersen MB, Nygaard R, Frimurer TM, Schwartz TW (2007) Activation of the CXCR3 chemokine receptor through anchoring of a small molecule chelator ligand between TM-III, -IV, and -VI. Mol Pharmacol 71(3): 930-941. 10.1124/mol.106.030031

[2] Kissow H, Hartmann B, Holst JJ, et al. (2012) Glucagon-like peptide-1 (GLP-1) receptor agonism or DPP-4 inhibition does not accelerate neoplasia in carcinogen treated mice. Regulatory peptides 179(1-3): 91-100. 10.1016/j.regpep.2012.08.016

[3] Kuhre RE, Ghiasi SM, Adriaenssens AE, et al. (2019) No direct effect of SGLT2 activity on glucagon secretion. Diabetologia 62(6): 1011-1023. 10.1007/s00125-019-4849-6

[4] Kuhre RE, Holst JJ (2019) Mechanisms Underlying Gut Hormone Secretion Using the Isolated Perfused Rat Small Intestine. JoVE(144): e58533. doi:10.3791/58533
